# Assessing the Spatiotemporal Variation in Distribution, Extent and NPP of Terrestrial Ecosystems in Response to Climate Change from 1911 to 2000

**DOI:** 10.1371/journal.pone.0080394

**Published:** 2013-11-25

**Authors:** Chengcheng Gang, Wei Zhou, Jianlong Li, Yizhao Chen, Shaojie Mu, Jizhou Ren, Jingming Chen, Pavel Ya. Groisman

**Affiliations:** 1 Global Change Research Institute, School of Life Science, Nanjing University, Nanjing,Jiangsu, P. R. China; 2 State Key Laboratory of Grassland Agro-ecosystems, College of Pastoral Agriculture Science and Technology, Lanzhou University, Lanzhou, Gansu, P. R. China; 3 Department of Geography, University of Toronto, Toronto, Ontario, Canada; 4 NOAA National Climatic Data Center, Asheville, North Carolina, United States of America; The Ohio State University, United States of America

## Abstract

To assess the variation in distribution, extent, and NPP of global natural vegetation in response to climate change in the period 1911–2000 and to provide a feasible method for climate change research in regions where historical data is difficult to obtain. In this research, variations in spatiotemporal distributions of global potential natural vegetation (PNV) from 1911 to 2000 were analyzed with the comprehensive sequential classification system (CSCS) and net primary production (NPP) of different ecosystems was evaluated with the synthetic model to determine the effect of climate change on the terrestrial ecosystems. The results showed that consistently rising global temperature and altered precipitation patterns had exerted strong influence on spatiotemporal distribution and productivities of terrestrial ecosystems, especially in the mid/high latitudes. Ecosystems in temperate zones expanded and desert area decreased as a consequence of climate variations. The vegetation that decreased the most was cold desert (18.79%), while the maximum increase (10.31%) was recorded in savanna. Additionally, the area of tundra and alpine steppe reduced significantly (5.43%) and were forced northward due to significant ascending temperature in the northern hemisphere. The global terrestrial ecosystems productivities increased by 2.09%, most of which was attributed to savanna (6.04%), tropical forest (0.99%), and temperate forest (5.49%). Most NPP losses were found in cold desert (27.33%). NPP increases displayed a latitudinal distribution. The NPP of tropical zones amounted to more than a half of total NPP, with an estimated increase of 1.32%. The increase in northern temperate zone was the second highest with 3.55%. Global NPP showed a significant positive correlation with mean annual precipitation in comparison with mean annual temperature and biological temperature. In general, effects of climate change on terrestrial ecosystems were deep and profound in 1911–2000, especially in the latter half of the period.

## Introduction

Studies regarding the interactions between global change and terrestrial ecosystems are becoming widespread in the current body of global change research. Increasing atmospheric CO_2_ concentration in the past decades has been accompanied by other global changes. Rising air temperatures and altered precipitation patterns are among the most prominent of the predicted changes that, along with elevated CO_2_, have affected ecosystem structure and function deeply and in a profound way [1], [2], [3]. Improving understanding of the interactions and feedback mechanisms of physical climate systems and environmental systems, predicting longer term trends, and preparing strategies for future events are grand challenges [4], [5]. Climate is the main driving force in the distribution of ecosystems, and vegetation is the most distinct indicator of this distribution [6]. Climate change affects vegetation mainly through changes in precipitation and temperature which affect the effective accumulated temperature and the content of soil organic matter [7], [8]. Net primary productivity (NPP), which measures the energy fixed by the plant community through photosynthesis and indicates the growth ability in a specific natural environment, provides a link between biomes and the climate system through the global carbon and water cycles. The dynamics of NPP can reflect the variations of ecosystems in response to climate change, which is of great significance to assess disturbance of terrestrial ecosystems and evaluate terrestrial carbon sink [9], [10].

Since the concept of PNV was introduced, many endeavors have been devoted to evaluating the impacts of simulated past and future climate change on ecosystems at regional-to-global scales [1], [11], [12], which greatly improves our ability to assess the interactions between terrestrial ecosystems and climate change. However, these models always require complicated parameters and input data to reflect the ecological processes and simulate the process of vegetation dynamics [13]. Additionally, the application of biogeographic models (e.g. Holdridge Life Zone) and equilibrium vegetation models (e.g. BIOME4) is mainly focused on forests. Based on the relationships of climate, soil and vegetation, CSCS was mainly driven by mean temperature and precipitation data which overcomes the deficiency of complicated and insufficient parameters, especially in regions that lack of collected data. After years of developed and optimized, the model has been widely used in terrestrial ecosystems classification and global change research [14], [15], [16], [17].

NPP refers to the organic matter that is fixed by plants mainly through the process of photosynthesis, and thus can reflect the growing status of vegetation and measure the amount of trophic energy flows in food webs and chains [18]. Vast research has been conducted to evaluate terrestrial NPP at multiple levels. NPP estimation models, such as climate-based models (i.e. MIAMI model [19], Thornthwaite Memorial model [19]), process-based models (e.g. CENTURY [1], TEM [20], BIOME-BGC [21]), and light use efficiency models (e.g. CASA [22], GLO-PEM [23]), have been widely reported, and their accuracy increased resulted from the significant development of remote sensing technology. However, parameters used in process-based models are complicated which leads to difficulties in data acquisition in some regions. This makes process-based models more suited for regional NPP estimation. In contrast, light use efficiency models are much more widely used in regional and global NPP estimation due to the readily available parameters that are derived from remote sensing data either directly or indirectly. However, satellite-based parameters employed in models, e.g. normalized difference vegetation index (NDVI), have only been accessible for the past 30 years which prevents their application in evaluating NPP over the length of a century. Although simple, climate-based models are valuable and quite capable of simulating global vegetation NPP and its variation in response to climate change over the length of a century when meteorological data is available [24]. In this paper, a synthetic model [25] was used to evaluate global vegetation NPP and its variations under climate change in the period of 1911–2000.

The interactions between terrestrial ecosystems and climate change ranges in timescale from seconds to millions of years and from local to worldwide in spatial scale. The structure and functions of vegetation are strongly determined by climate change primary in terms of temperature and precipitation [26], [27], yet the bidirectional influences of climate change and terrestrial vegetation are still obscure [28]. To better clarify this problem, in this paper, the dynamics of spatiotemporal distribution, extent, and NPP of global terrestrial ecosystems from 1911 to 2000 were quantitatively assessed using the CSCS and synthetic model. The correlation between NPP dynamics and climate factors in the same period was also studied to investigate its response to climate change. The results of this work provided a general outlook of the effects of climate change on terrestrial ecosystems in the past century, and the outcomes may complement the IPCC report. Furthermore, methods used in this paper can serve as a guide for studies in past and future global change in regions lacking collected data.

## Materials and Methods

### Global climatic data

In this paper, the global climate dataset CRU_TS 2.1 from the climate research unit (CRU) (http://www.cru.uea.ac.uk/~timm/grid/CRU_TS_2_1.html) was empolyed in the CSCS to generate global PNV maps and in the synthetic model to simulate NPP. The dataset of grids extends from 1901 to 2002, covers the global land surface (excluding Antarctica) at a 0.5° resolution, and provides best estimates of month-by-month variations in climate variables. The well-established dataset has already been widely applied [29]. In this study, the mean annual temperature (MAT) and mean annual precipitation (MAP) data in the period 1911–2000 were incorporated from monthly grid data using ArcGIS v9.3 software (ESRI, Redlands, CA, USA). Additionally, the Mollweide projection with the WGS_1984 spheroid was applied to all of the related databases for the calculation of the area of vegetation types.

### The CSCS model

Based on hydrothermal conditions, the CSCS model is composed of three levels: class, subclass, and type. The class level, the basic unit, is determined by bioclimatic conditions, the subclass level is classified by edaphic conditions, and the type level is based on vegetation characteristics [15]. Subclasses are integrated into classes according to an index of moisture and temperature which captures the natural occurrence of vegetation ecosystems. The classes are mainly established by annual cumulative temperature above 0°C (Σ*θ*) (Growing Degree-Days on 0°C base, GDD0) and humidity index (*K*), as calculated by:
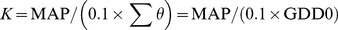
(1)where MAP is the mean annual precipitation (mm) and 0.1 is an empirical parameter. To more explicitly reflect the spatial distribution of PNV at a global scale, classes were regrouped into 10 vegetation types, i.e. tundra &alpine steppe, cold desert, semi-desert, steppe, temperate humid grassland, warm desert, savanna, temperate forest, subtropical forest, tropical forest.

### Global potential natural vegetation maps

To simulate the dynamics of PNV more reasonably, the global biomes maps were produced at 30-year intervals. According to the IPCC Third Assessment Report, the periods that most obviously increased in temperature during the 20th century were from 1910 to 1945 and 1976 to 2000 [30]. We divided these 90 years into three intervals as 1911 to 1940 (T1), 1941 to 1970 (T2), and 1971 to 2000 (T3).

### NPP model

NPP of natural vegetation was simulated using the synthetic model. The model was based on actual evapotranspiration which was closely related to the photosynthesis of vegetation, and was established mainly on the biomass data from 125 sets of natural mature forest in China and 23 sets of natural vegetation NPP that included forest, grassland, and desert. These data were obtained during the International Biological Program (IBP) by Efimova [31], [32]. This model integrated the interaction among many variables and was developed in light of the same references as the Chikugo model [33]:

NPP
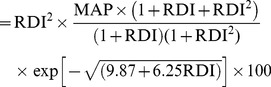
(2)

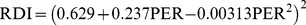
(3)

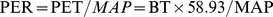
(4)where MAP is the mean annual precipitation (mm), RDI is the radioactive dryness index which can be calculated by PER, PER is the rate of evapotranspiration, PET is potential evapotranspiration (mm), and BT is biological temperature which is the average temperature during the vegetative growth of plants (0∼30°C, temperatures below 0°C and above 30°C are excluded). NPP is calculated in units of g DW m^−2^ yr^−1^.

### Correlation between NPP and climate factors

The Pearson correlation coefficient was employed to reflect the relationship between NPP and climate factors, including MAP, MAT and BT. The spatial distribution maps of correlation coefficients between NPP and climate factors were obtained through the equation of correlative analysis, which is expressed as follows:
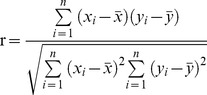
(5)where *yi* refers to climate factors (including MAP (mm), MAT (°C), and BT (°C)) in year *i*; and 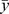
represents the mean climate values over the years. When the correlation coefficient was tested for significance (*P<0.01* or *P<0.05*), it displayed an “extremely significant” or “significant” linear correlation [34].

## Results

### Changes of climate factors during 1911–2000

**Figure 1:**
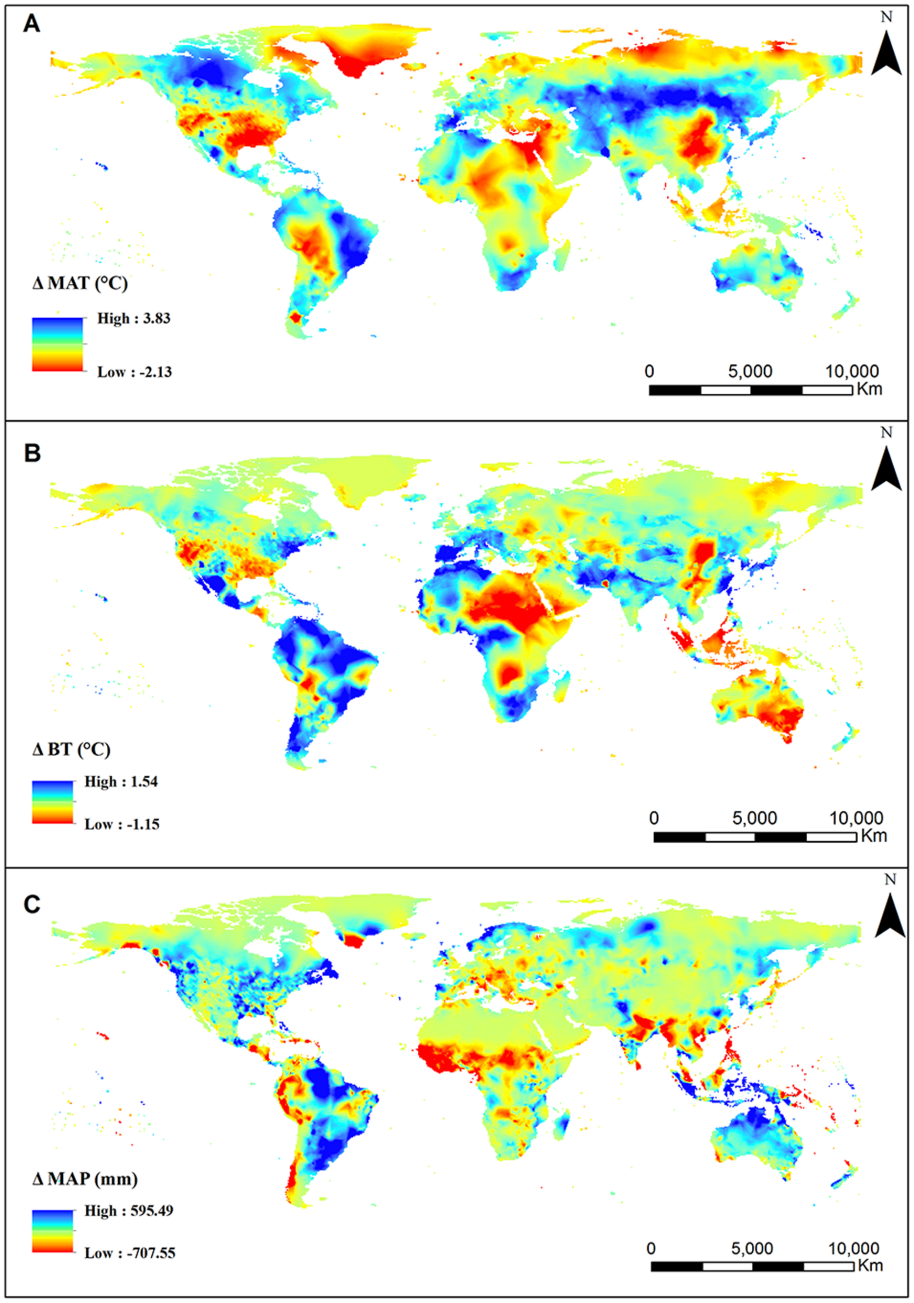
Dynamic of climate factors in the period 1911–2000. The variations of climatic variables were derived from CRU_TS 2.1 data. (a): mean annual temperature (MAT), (b): biological temperature (BT), (c): mean annual precipitation (MAP).

Structure and function of ecosystems are strongly determined by climate influences, primarily through temperature ranges and precipitation available. According to our research, the global warming showed an obvious zonal distribution, especially in the mid- and high latitude on northern hemisphere (Figure.1). Based on our findings, for the past 90 years extending from 1911 to 2000, the global MAT increased by 0.23°C. Regions that showed a decreasing trend amounted to 23.12% of total land area, and were mainly distributed in the south of Greenland, Mideast and east of America, west of Brazilian plateau, the Mediterranean Coast, Yunnan-Guizhou Plateau, part of the Qinghai-Tibet Plateau, and the north of Siberia. Accordingly, the global BT moderately increased by 0.06°C in the 90 years, which was closely related to the continuously increasing global temperature. 65.2% of the total land presented an increasing trend, while regions with decreased BT were mainly located in the east and southeast of the Sahara, southeast of Australia, and the Mongolian Plateau. With regards to MAP, a distinct spatial heterogeneity was observed with an overall 14.39 mm increase globally. Regions that showed increasing MAP were estimated to be 65.57% of total land area during T3 period relative to T1 period. This increase was mainly located on both sides of equator and mid- and high latitudes while regions with reduced precipitation mainly occurred 10° north of the equator and on west coast of South America. (The dataset of MAT and MAP for each of the three periods are available in the Appendix S1– S6)

### Shifts of terrestrial ecosystems on the basis of the CSCS in 1911–2000

**Figure 2:**
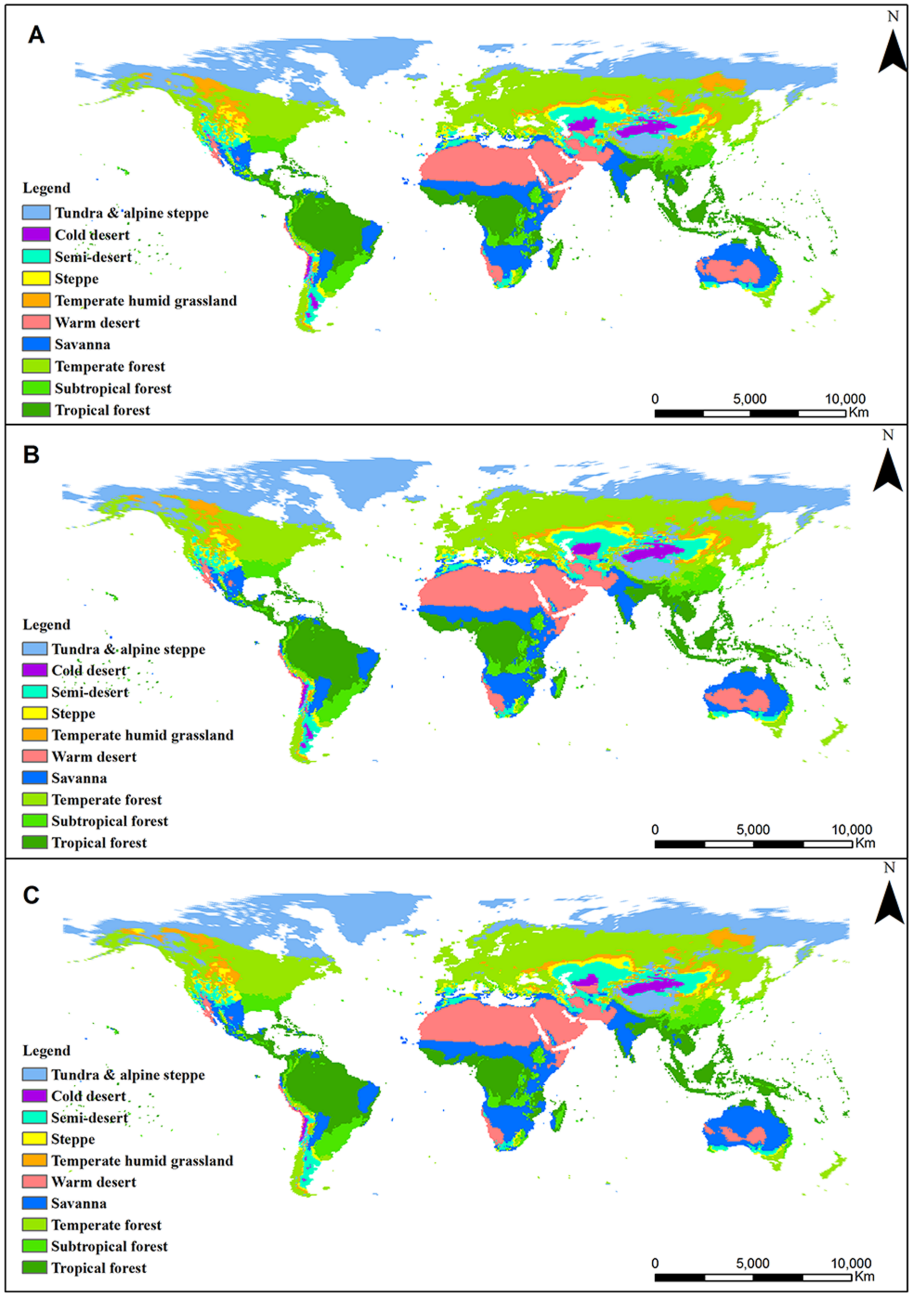
Spatial distribution of global natural vegetaiton biomes in the period 1911–2000. (a): T1 period (1911–1940), (b): T2 period (1941–1970), (c): T3 period (1971–2000). The different colors represent the different ecosystems, tundra & alpine steppe (12.85%), cold desert (1.56%), warm desert (13.93%), semi-desert (5.96%), savanna (17.66%), steppe (3.29%), temperate humid grassland (5.86%) and Forest (39.65%) in T3 period.

The global PNV maps of the T1, T2, and T3 periods were obtained through the CSCS. As indicated in Figure.2, the maps showed an obvious zonal distribution of terrestrial ecosystems. Tundra & alpine steppe were mainly distributed in the high latitudes and elevations of the northern hemisphere, in places such as Siberia, Greenland, the north of North America, and the Qinghai-Tibet Plateau. In the three desert types, the cold desert was restricted to the northwest of China, part of the Turanian Plateau, and scattered areas along the Andes. The semi-desert encircled the cold desert in Central Asia, Mongolia, and the Brazilian Plateau, while the warm desert was mainly localized in the Sahara Desert, the Arabian Peninsula, and central Australia. The steppe was distributed in areas neighboring the semi-desert, which mainly located in Inner Mongolia, part of West Asia, and the Great Plains of America. Temperate humid grassland was mainly localized in Canada and Eurasia adjacent to steppe. Savanna, strongly controlled by tropical savanna climate which is highly temperate and distinguished by a dry and wet season, was mainly distributed south and north of rainforest in Africa and South America and encircling warm desert in Australia. Most of the temperate forest was distributed near 60 degrees north in Asia and Europe, the east of North America, and only a little in Oceania. Subtropical forest was mainly localized in the southeast of North America and China, while tropical forest was mainly located on both sides of the equator. (The global terrestrial biomes simulated by CSCS of the three periods are available in the Appendix S7–S9)

Climate change is the main driver of the patterns and processes in global ecosystems over long time periods, and determined the succession of different vegetation types. At a global scale, the area of tundra & alpine steppe decreased significantly with 96.5×10^4^ km^2^ (5.43%) in the period of 1911–2000 (Table 1). Similarly, all three desert vegetation types shrunk during the same period, semi-desert, cold desert and warm desert decreased by 1.55%, 18.79%, and 6.02%, respectively. The persistently increasing area was found in savanna with a small increase from T1 to T2 (0.90%) and a marked increase from T2 to T3 (9.32%). With regards to steppe and temperate humid grassland, both decreased greatly by 9.68% and 11.36% from the T1 to T2 periods, respectively, and then increased moderately from the T2 to T3 periods (1.00% and 2.53%). Temperate forest expanded by 4.96%, tropical forest rose by just 0.17%, and subtropical forest decreased by 0.94%. Areas of different ecosystems on continental levels are shown in Table 1.

**Table 1:** Areas of terrestrial ecosystems at continental levels in 1911–2000.

	Africa	Asia	Europe	Oceania	North America	South America	Global
Tundra & alpine steppe	×	1933.87±31.36	311.27±6.38	×	1793.97±61.70	23.73±11.05	4089.27±84.72
		(−1.00%)	(2.02%)		(−6.64%)	(−63.76%)	(−3.92%)
Cold desert	×	114.01±11.42	×	×	1.53±1.19	351.41±18.34	139.65±23.57
		(−16.93%)			(−86.82%)	(−9.95%)	(−27.33%)
Semi-desert	300.77±20.33	1495.03±53.91	149.23±22.71	265.22±13.33	502.37±32.94	226.49±18.05	3182.06±35.76
	(−12.66%)	(2.82%)	(33.03%)	(−2.02%)	(−11.98%)	(−14.38%)	(−0.15%)
Steppe	185.41±18.41	831.08±46.67	342.73±24.62	102.19±11.16	554.81±45.80	66.58±5.54	2437.90±121.60
	(−16.46%)	(−0.40%)	(−10.44%)	(−17.34%)	(−13.35%)	(−11.64%)	(−8.35%)
Temperate humid grassland	×	920.62±61.97	200.47±9.65	×	618.30±22.62	9.21±0.99	1809.96±82.44
		(−7.12%)	(−4.86%)		(−4.54%)	(−19.12%)	(−6.22%)
Warm desert	490.15±13.36	293.02±9.74	×	424.10±120.68	42.87±8.49	2471.83±90.92	1304.57±136.04
	(5.25%)	(−0.66%)		(−42.48%)	(−3.94%)	(−4.21%)	(−17.78%)
Savanna	8715.89±195.12	2400.37±247.63	42.08±6.96	3012.38±546.33	1214.97±36.09	772.24±35.37	19114.76±950.51
	(4.19%)	(18.44%)	(30.18%)	(36.71%)	(4.51%)	(−8.76%)	(9.42%)
Temperate forest	105.88±17.18	5010.41±250.77	4487.59±45.10	582.62±18.20	4895.13±358.26	2991.46±109.50	17726.47±503.00
	(−26.48%)	(5.97%)	(1.53%)	(−1.52%)	(15.77%)	(2.90%)	(5.49%)
Subtropical forest	2815.76±160.11	3429.22±48.04	82.33±10.01	283.91±15.98	1989.43±14.98	18044.70±412.52	12631.45±108.87
	(−6.14%)	(−0.79%)	(21.90%)	(1.83%)	(1.04%)	(4.50%)	(−0.70%)
Tropical forest	10375.76±451.62	10736.07±234.12	×	735.28±73.55	1757.00±28.69	24985.32±355.37	50001.87±531.72
	(−5.21%)	(−1.39%)		(20.07%)	(2.53%)	(2.73%)	(0.99%)

(Unit: × 10^4^ km^2^) “×” indicates that a continent did not have a type of vegetation; a negative sign within parentheses indicates a decreasing trend in the period 1911–2000.

### Validation of synthetic model

**Figure 3:**
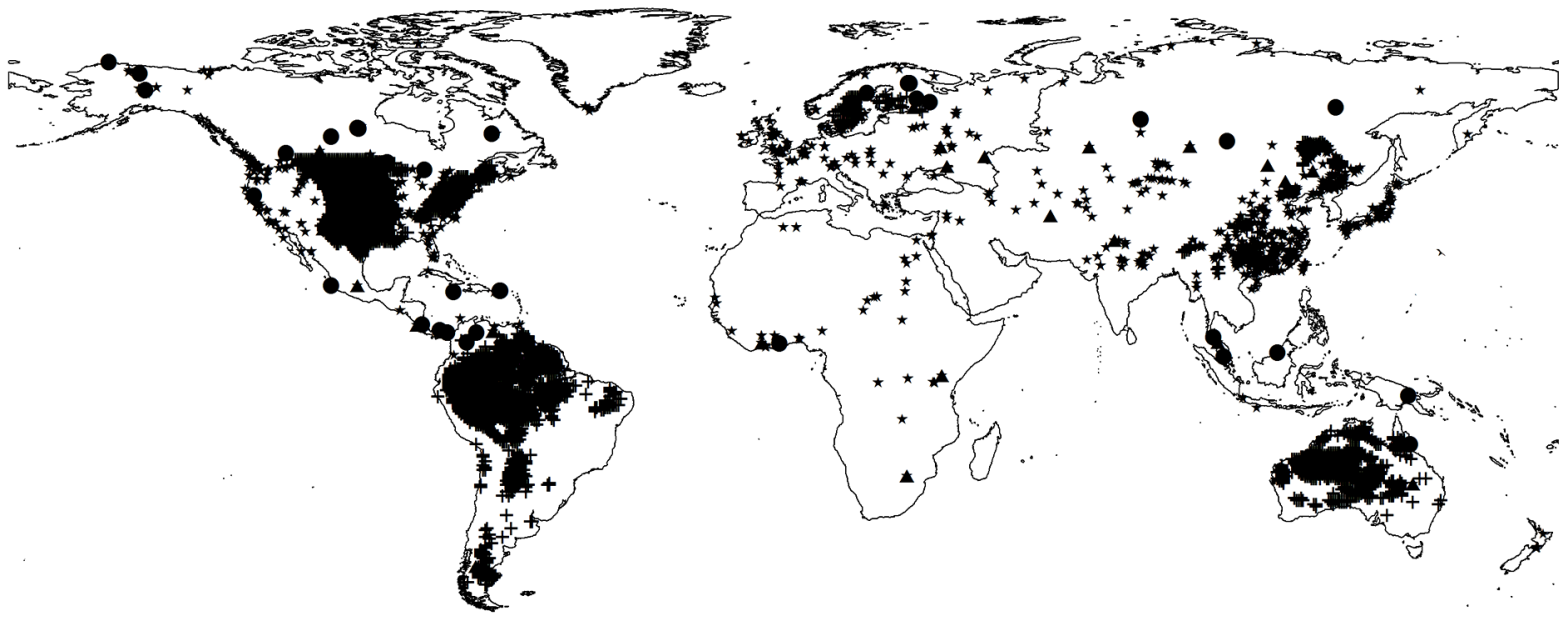
Map showing the geographical distribution of detailed terrestrial NPP study sites. The dataset are obtained from the Oak Ridge National Laboratory (ORNL), Distributed Active Archive Center database. • =  grassland sites included in the present analysis; ▴ =  NPP study sites forests (tropical forest, temperate forest and boreal forest), ★ and + =  multi biomes – EDMI (B and C) data [63]. These data, and further information about the study sites, are publicly available at www.daac.ornl.gov/NPP/.

**Figure 4:**
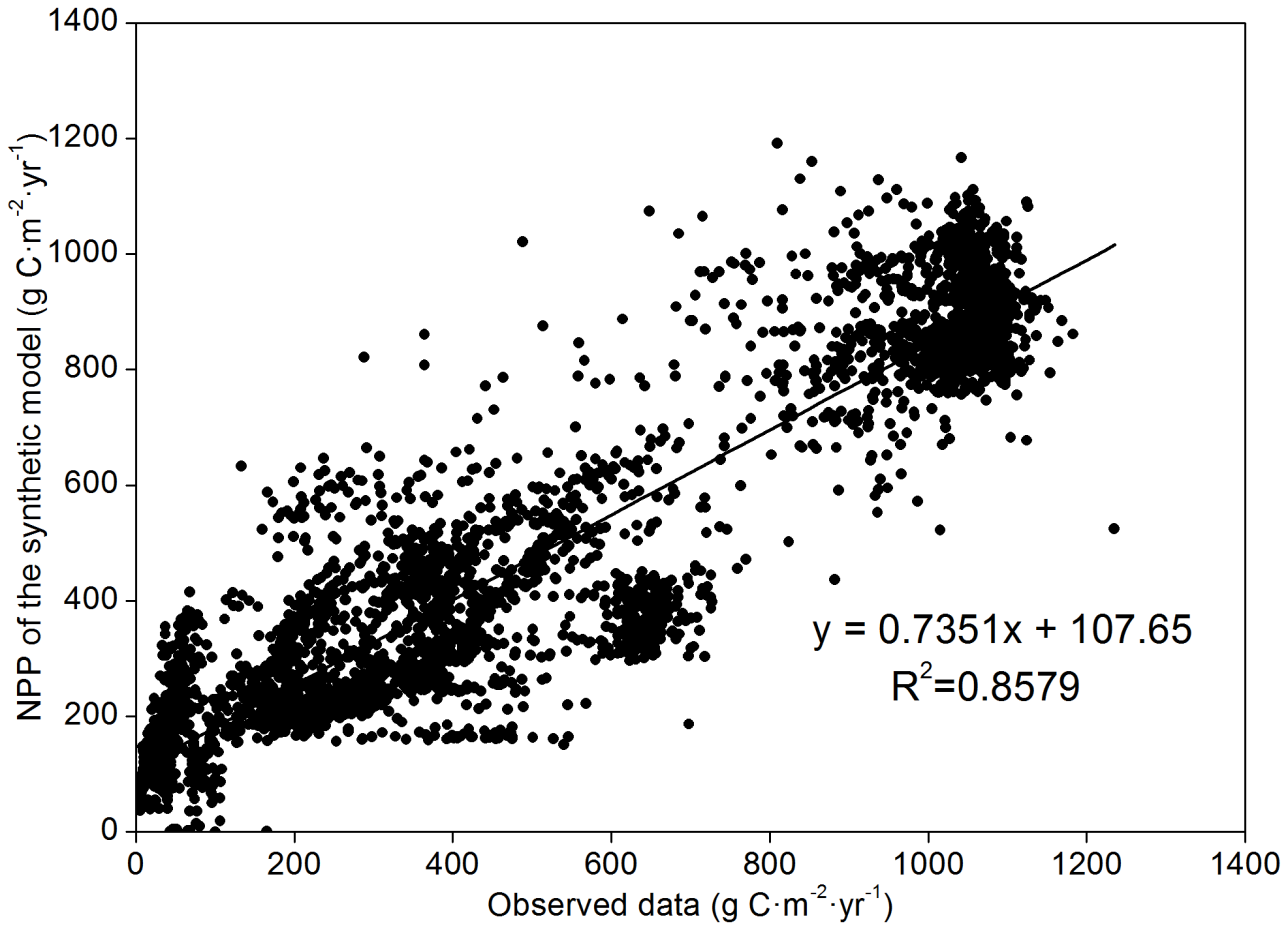
Comparison of NPP value simulated by synthetic model and observed data (*r = 0.9262, P<0.001*). The observed data are collected from the Oak Ridge National Laboratory (ORNL). These data, and further information about the study sites, are publicly available at www.daac.ornl.gov/NPP/.

The simulated total NPP of the synthetic model was compared with current available data according to Ito's search [35], and the gridded simulated value was also compared with observed data. In this research, the majority of observed NPP data was gathered from the Oak Ridge National Laboratories (ORNL) Net Primary Production database (http://daac.ornl.gov/NPP/npp_home.shtml) [36], which is especially useful for model and hypothesis testing. These study sites represent a broad range of vegetation types as defined by eco-regions or climatic zones [37], [38]. Data were mapped according to their associated geographic coordinates, and sites with incomplete geographic coordinates or absence of total NPP data were excluded from the study (Figure.3). Based on Table 2 and Figure.4, we can see that the simulated NPP of the synthetic model is in good agreement with available published and observed data (R^2^ = 0.8579). Therefore, the synthetic model is capable of evaluating global terrestrial ecosystems productivities and their variations over the length of a century.

**Table 2:** Comparison of published values of present terrestrial net primary productivity (NPPT) and our research.

Reference	NPPT (Pg·C·yr^–1^)
Woodwell et al. [39]	52.8
IPCC 1st Assessment Report [40]	52
Siegenthaler & Sarmiento [41]	51.97
Sundquist [42]	60
IPCC 2nd Assessment Report [43]	61.3
Schlesinger [44]	60
Schimel et al. [45] after Cramer et al. [46]	42–68
Geider et al. [47]	56.4
IPCC 3rd Assessment Report [48]	60
Gruber et al. [49]	57
**Our search**	**50.78**

### Changes of global vegetation NPP

Climate change is expected to affect terrestrial ecosystems' NPP through disturbing structures, functions, energy flows, etc. To understand the effect of climate change on ecosystem production, the NPP was calculated using the synthetic model.

At a global scale, the total NPP of terrestrial ecosystems increased gradually by 2317.97 Tg DW yr^−1^ (2.09%) over the period of 1911–2000 (Table 3). This was mainly attributed to the increase of savanna (1740.14 Tg DW yr^−1^, 9.42%), temperate forest (942.08 Tg DW yr^−1^, 5.49%), and tropical forest (489.12 Tg DW yr^−1^, 0.99%). The NPP of forest vegetation accounted for more than 70% of the global NPP. The maximum estimation was in tropical forest. In contrast to the increasing trend of temperate forest and tropical forest, subtropical forest presented a slightly descending trend of 0.70%. With regards to grasslands vegetation, the NPP of savanna, which amounted to nearly 70% of total grasslands NPP, was found to be the largest NPP increase. The NPP of tundra & alpine steppe, steppe, and temperate humid grassland all declined, by 162.78 Tg DW yr^−1^ (3.92%), 197.17 Tg DW yr^−1^ (7.65%), and 118.40 Tg DW yr^−1^ (6.22%), respectively. The productivities of desert types were relatively low, occupying less than 5% of total NPP. The NPP losses were higher in cold desert (27.33%) than in warm desert (17.78%), while NPP of semi-desert was basically flat with a slight increase (0.15%) in the same period. Continental distributions of terrestrial NPP are shown in Table 3.

**Table 3:** NPP of terrestrial ecosystems at continental levels in 1911-2000.

	Africa	Asia	Europe	Oceania	North America	South America	Global
Tundra & alpine steppe	×	1933.87±31.36	311.27±6.38	×	1793.97±61.70	23.73±11.05	4089.27±84.72
		(−1.00%)	(2.02%)		(−6.64%)	(−63.76%)	(−3.92%)
Cold desert	×	114.01±11.42	×	×	1.53±1.19	351.41±18.34	139.65±23.57
		(−16.93%)			(−86.82%)	(−9.95%)	(−27.33%)
Semi-desert	300.77±20.33	1495.03±53.91	149.23±22.71	265.22±13.33	502.37±32.94	226.49±18.05	3182.06±35.76
	(−12.66%)	(2.82%)	(33.03%)	(−2.02%)	(−11.98%)	(−14.38%)	(−0.15%)
Steppe	185.41±18.41	831.08±46.67	342.73±24.62	102.19±11.16	554.81±45.80	66.58±5.54	2437.90±121.60
	(−16.46%)	(−0.40%)	(−10.44%)	(−17.34%)	(−13.35%)	(−11.64%)	(−8.35%)
Temperate humid grassland	×	920.62±61.97	200.47±9.65	×	618.30±22.62	9.21±0.99	1809.96±82.44
		(−7.12%)	(−4.86%)		(−4.54%)	(−19.12%)	(−6.22%)
Warm desert	490.15±13.36	293.02±9.74	×	424.10±120.68	42.87±8.49	2471.83±90.92	1304.57±136.04
	(5.25%)	(−0.66%)		(−42.48%)	(−3.94%)	(−4.21%)	(−17.78%)
Savanna	8715.89±195.12	2400.37±247.63	42.08±6.96	3012.38±546.33	1214.97±36.09	772.24±35.37	19114.76±950.51
	(4.19%)	(18.44%)	(30.18%)	(36.71%)	(4.51%)	(−8.76%)	(9.42%)
Temperate forest	105.88±17.18	5010.41±250.77	4487.59±45.10	582.62±18.20	4895.13±358.26	2991.46±109.50	17726.47±503.00
	(−26.48%)	(5.97%)	(1.53%)	(−1.52%)	(15.77%)	(2.90%)	(5.49%)
Subtropical forest	2815.76±160.11	3429.22±48.04	82.33±10.01	283.91±15.98	1989.43±14.98	18044.70±412.52	12631.45±108.87
	(−6.14%)	(−0.79%)	(21.90%)	(1.83%)	(1.04%)	(4.50%)	(−0.70%)
Tropical forest	10375.76±451.62	10736.07±234.12	×	735.28±73.55	1757.00±28.69	24985.32±355.37	50001.87±531.72
	(−5.21%)	(−1.39%)		(20.07%)	(2.53%)	(2.73%)	(0.99%)

(Unit: Tg DW yr^−1^). “×” indicates that a continent did not have a type of vegetation; a negative sign within parentheses indicates a decreasing trend in the period 1911–2000.

**Figure 5:**
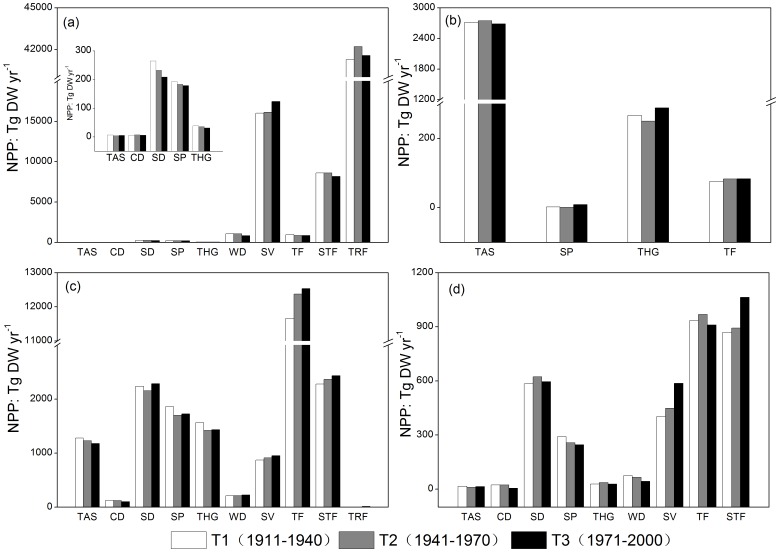
Variations of terrestrial ecosystems NPP in climatic zones in the period 1911–2000. (a): Tropical zone, (b): North frigid zone, (c): North temperate zone, (d): South temperate zone. South frigid zone was not involved in this research. TAS: tundra & alpine steppe; CD: Cold desert; SD: Semi-desert; SP: Steppe; THG: Temperate humid grassland; WD: warm desert; SV: savanna; TF: Tempearte forest; STF: Subtropical forest; TRF: Tropical forest.

From a climatic regions perspective, vegetation NPP in the tropical region (Figure. 5(a)), which amounted to more than half of total terrestrial ecosystem NPP, increased slightly by 1.32% in the period 1911–2000. The NPP of tropical forest and savanna took up nearly 60% and 25%, respectively, of that tropical region. The NPP of savanna in this period increased remarkably by 1441.82 Tg DW yr^−1^ (8.99%), while the increasing trend of tropical forest (0.70%) was relatively low. In contrast, a declining trend of NPP was observed in other ecosystems. For instance, the decrease in warm desert was remarkable (21.40%). The NPP of ecosystems in the northern frigid zone also increased slightly overall (0.16%) over the course of the entire study period (Figure. 5(b)). Temperate humid grassland was estimated to increase by 21.21 Tg DW yr^−1^ (7.95%). The NPP of steppe and temperate forest also went up by 7.13 Tg DW yr^−1^ and 6.81 Tg DW yr^−1^, respectively. A slight decrease (1.11%) was observed in tundra & alpine steppe, the NPP of which occupied nearly 90% of vegetation NPP in the northern frigid zone. As can be seen in Figure 5 (c) and (d), the NPP in the northern temperate zone is nearly seven times that of the southern temperate zone, and both increased gradually in this period by 3.55% and 8.27%, respectively. In the northern temperate zone, the temperate forest NPP amounted to more than 50% of the total and contributed the most to the NPP increase. The increasing trend of NPP was also found in savanna (9.50%), subtropical forest (6.82%), and semi-desert (2.18%), whereas a declining trend was observed in tundra & alpine steppe (7.89%), cold desert (18.47%), steppe (7.42%), and temperate humid grassland (8.42%). In the southern temperate zone, NPP of subtropical forest and savanna went up by 193.14 Tg DW yr^−1^ (22.19%) and 183.76 Tg DW yr^−1^ (45.64%), respectively. Similarly, NPP of semi-desert and temperate humid grassland displayed an increasing tendency with 2.03% and 2.11%, respectively. In contrast, NPP losses were found in tundra & alpine steppe (2.53%), cold desert (19.04%), steppe (15.50%), warm desert (42.15%) and temperate forest (2.65%). (The dataset of global terrestrial NPP based on the synthetic model of the three periods are available in the Appendix S10–S12)

### Correlations between NPP and climate factors

**Figure 6:**
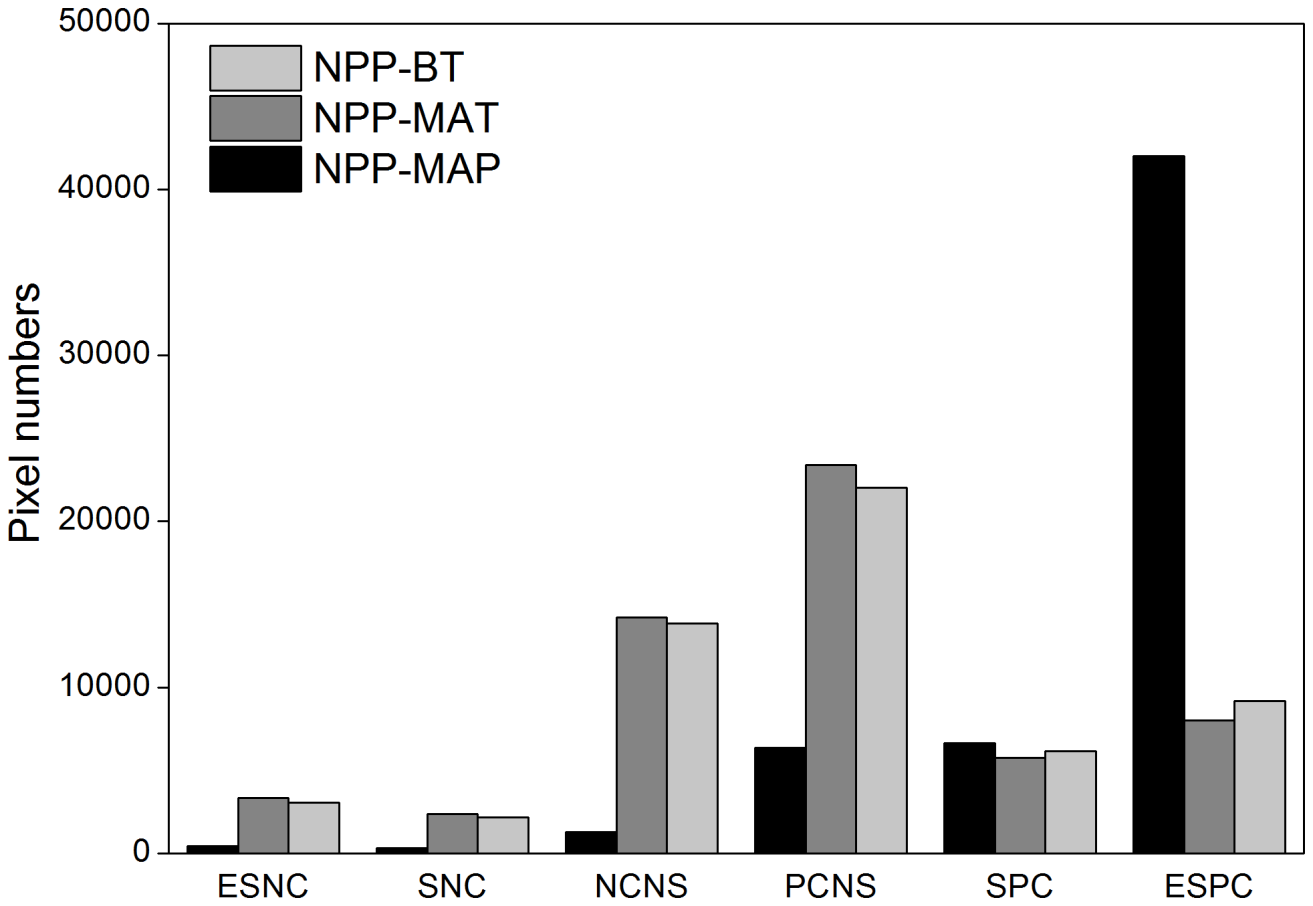
Proportion of different correlations between global NPP and MAP, MAT and BT. ESNC is extremely significant negative correlation; SNC is significant negative correlation; NCNS is negative correlation but none significant; PCNS is positive correlation but none significant; SPC is significant positive correlation; ESPC is extremely significant positive correlation.

The maps of the spatial distribution of correlation coefficients were obtained based on the correlation coefficients between NPP and climatic factors from 1911 to 2000. In this period, the positive correlation between NPP and MAP predominated in the world (Figure 6). 73.6% of regions showed extremely significant positive correlation, and the global average correlation coefficient was 0.88. Regions displaying a negative correlation were mainly located in Alaska and some arid and semiarid areas. In contrast, the correlation between NPP and MAT showed great spatial heterogeneity, especially in northern hemisphere. Regions that displayed a positive correlation occupied 65.12% of the total area and were mainly distributed in north of North America, east of Africa, and most areas in Australia. Globally, regions with a correlation lower than the significance level of 0.05 accounted for 34.10%. The correlation between NPP and BT was also analyzed in this paper. Based on our findings, regions that showed positive correlations made up 66.24%. Regions presenting insignificant correlation between NPP and BT/MAT were estimated to be 63.58% and 65.9% of total land covers, respectively. This implies that at global scale, vegetation productivities were more affected by precipitation than MAT/BT, but at regional scales, the significance of MAT and BT are highlighted.

## Discussion

### Discussion of the methodology

The meteorological dataset used in this paper was CRU TS 2.1, a well-documented dataset with over a century-long time scale and a high-resolution (0.5) latitude-longitude grid with global coverage. Such grids may be inappropriate for regional studies but are more useful for larger scales. The gridded data was constructed from the global network of meteorological observation stations, mainly obtained from seven sources [29], [50]. The station network for CRU variables exhibited a gradual increase in the total number of stations from 1901 to about 1980. Distributions of precipitation stations are continentally extensive with high density in America, Australia, Europe, and eastern Asia and low density in Siberia and central Asia. There were almost half as many precipitation stations as mean temperature stations. Most of the mean temperature stations were distributed in America, Europe, and eastern Asia. Records from stations were checked and merged using the overlap function and if the records were not overlapped, a fixed reference series was constructed which is one of the main error sources of CRU data. It was indicated that side effects emerge when examining long-term average statistical properties or the frequency of extreme events using a fixed baseline period (1961–1990) [29]. The other source of error may lie in the homogenization method. Due to the need of checking for inhomogeneities, records obtained from areas and periods when density is low were added to the database without any checks. In addition, the interpolation method is also a weakness in detecting abrupt rather than gradual inhomogeneities in the data which cannot be detected unless they are widespread [51]. However, the error of the CRU dataset is substantially smaller than the temperature trends believed to have been occurring during the twentieth century, so it is capable of correctly reflecting climate change.

The natural vegetation maps of the three intervals in 1911–2000 were obtained through the CSCS. The CSCS was established by linking vegetation with their climatic and edaphic factors [15]. Humidity index, determined by MAP and cumulative temperature, is the main parameter in the CSCS which presents promising applications in the research of simulating past and future global change, especially in regions where lack collected data. However, this system does not take the effects of underlying surfaces into account which lowers its accuracy in regions with complicated underlying surfaces such as mountain regions. In high latitude and elevation regions, it also underestimates the precipitation data due to neglecting the supply of underground water and melt water, thereby increasing the errors and bias. Although there are errors in assessment, the methodology enables a novel method of natural vegetation classification and easily demonstrates the spatial zonal distribution and dynamics of vegetation systems in various climate conditions at a global scale. Therefore, the CSCS is a feasible approach to map the global biomes and their response to climate change over the length of a century.

In this study, we employed a synthetic model to simulate terrestrial ecosystems' NPP during 1911–2000. Based on the water use efficiency of vegetation, determined by the ratio of the CO_2_ flux equation to vapor flux equations, the synthetic model is an actual evapotranspiration model which provides the connection between water balance and heat balance, and reflects the effects of energy and water on the rate of evaporation. Because this model mainly focused on the effect of water and heat on plant ecophysiology, it does not take the effects of CO_2_ concentration, soil nutrients, and interactions between vegetation and climate systems on NPP into consideration. Nevertheless, due to its sound mechanism and easily available data, the synthetic model is capable of detecting global NPP and variations in response to long term climate change. The synthetic model has been widely used in terrestrial ecosystems NPP estimation with techniques that are simpler, yet still useful for many cases [52], [53].

### Effects of climate change on distributions, extent and NPP of terrestrial ecosystems

This study demonstrated a comprehensive 90-year examination of the spatiotemporal variation in global terrestrial ecosystems and NPP based on the CSCS model and synthetic model. The simulated results showed that climate change played a crucial role in influencing the spatiotemporal distribution and NPP of terrestrial ecosystems.

The simulated results indicated that ecosystems were experiencing a gradual and irreversible change under climate change with an expansion of forest and savanna and a shrinking of tundra & alpine steppe, steppe, and temperate humid grassland [54]. As a result of climate change, vegetation in temperate zones was pushed to high elevations and latitudes which forced a readaptation to the environment and a reduction in productivities. Ecosystems in mid-and high latitudes in the northern hemisphere were more vulnerable and sensitive to climate change, and opportunities for these ecosystems to adapt to changes were limited because these systems react most strongly to globally induced climate change [30]. We found that the area of tundra & alpine steppe declined by approximately 88.0×10^4^ km^2^ from 1911 to 2000 globally. The south edge showed a persistent movement northward and eastward, and its current extent is likely to be encroached by grassland or forest [55]. Vegetation in semiarid areas, such as steppe, seemed to be replaced by vegetation accommodated to arid zones or sub-humid zones, e.g. semi-desert, savanna [4]. In Australia, shifts in rainfall patterns favored the establishment of woody vegetation e.g. woody savanna, and encroachment of warm desert [56], [57].

According to the global NPP derived from the synthetic model, most of the productivities were attributed to tropical forest, savanna, and temperate forest, and nearly 60% was estimated to occur in the tropical regions [58], [59]. In the period 1911–2000, the global NPP increased gradually by 2317.97 Tg DW yr^−1^. Most of this increase resulted from the increasing trend of savanna (9.42%), tropical forest (0.99%), and temperate forest (5.49%). This trend was also captured by the research of Cramer et al. [60] in which six dynamic global vegetation models were used to evaluate global terrestrial ecosystems in response to CO_2_ and climate change. Wang et al. [61] reported that the obvious climatic warming in the northern temperate regions had led to the general increase of temperate forest NPP from 1901 to 2009. In tropical regions, the NPP of tropical forest showed a slight increase of 0.70% [62]. The NPP of savanna increased by 8.99%. In Africa, warm desert and savanna expanded in some arid and semi-arid regions which increased productivities. However, high temperature also resulted in strong evapotranspiration which led to NPP decrease in some regions [63], [64]. Grace et al. [65] also reported similar results of tropical savanna in Australia, India, and South America. In northern frigid zones, rising temperature led to the decrease of the NPP of tundra & alpine steppe. In contrast, the expansion of steppe and temperate humid grassland resulted in an eventual increase in their NPP [66]. In temperate regions, temperate forest was the dominant ecosystem both in northern and southern temperate zones. However, we found a positive trend of NPP in northern temperate forest with 7.42%, whereas in southern temperate forest, NPP went up in the T2 period (3.59%) but went down in T3 period (6.02%) [67], [68], [69]. The reason for this may lie in the fact that during the 20th century, besides the obvious rising temperature in northern mid-latitude, precipitation in much of the northern hemisphere also displayed an increasing trend by 0.2% to 1% per decade. However, no similar variations were observed in the southern hemisphere [30]. All these variations may be a sign that the late 20^th^ century is a critical turning point for the significant change of terrestrial ecosystems as a consequence of global climate change.

From a global perspective, the positive correlation between NPP and MAP was more obvious than that of MAT and BT, indicating that precipitation was the most important climatic factor determining the productivities of vegetation. This is in agreement with previous studies [48], [70]. Recent global warming had extended vegetation growing seasons in most regions [71], [72] which led to the increase of biological temperature for plant growth. Variations in MAT would exert a more significant influence on NPP at a regional scale [73]. Zhang et al. [52] reported that in the last 50 years NPP in Inner Mongolia showed a positive correlation (R^2^ = 0.64) with temperature variations.

The overall conclusion from this analysis is that a range of impacts on global terrestrial ecosystems and NPP were observed under climate change from 1911 to 2000 and effects varied greatly among different ecosystems. Ecosystems in mid- and high latitudes, e.g. tundra & alpine steppe, were forced to adapt to a new habitat, whereas, the area of savanna, temperate forest, and tropical forest all increased. The past 90 years, particularly the latter half of last century, also witnessed an increasing trend of global NPP. Most of this increase was attributed to tropical forest, temperate forest, and savanna. NPP increase displayed a latitudinal distribution, particularly in tropical zones and northern temperate zones. In addition, there would be an increased chance for forest and grassland degradation from wild fires and invasive species as a result of global warming and reduced precipitation. In summary, although terrestrial productivities increased under climate change in the period 1911–2000, some ecosystems benefitted from it but others were negatively affected. Some regions were undergoing significant and irreversible change, and these effects may continue.

## Supporting Information

Appendix S1 **MAP in T1 period.**
(TIF)

Appendix S2 **MAP in T2 period.**
(TIF)

Appendix S3 **MAP in T3 period.**
(TIF)

Appendix S4 **MAT in T1 period.**
(TIF)

Appendix S5 **MAT in T2 period.**
(TIF)

Appendix S6 **MAT in T3 period.**
(TIF)

Appendix S7 **The simulated global vegetation in T1 period.**
(TIF)

Appendix S8 **The simulated global vegetation in T2 period.**
(TIF)

Appendix S9 **The simulated global vegetation in T3 period.**
(TIF)

Appendix S10 **Global NPP in T1 period.**
(TIF)

Appendix S11 **Global NPP in T2 period.**
(TIF)

Appendix S12 **Global NPP in T3 period**.
(TIF)
