# Correction: Assessing the Spatiotemporal Variation in Distribution, Extent and NPP of Terrestrial Ecosystems in Response to Climate Change from 1911 to 2000

**DOI:** 10.1371/journal.pone.0106175

**Published:** 2014-08-18

**Authors:** The PLOS ONE Staff

The corresponding author, Jianlong Li, has provided an additional email address: jianlongli@gmail.com

There are errors in the Funding section. The correct funding information is as follows: This work was supported by "The Key Project of Chinese National Programs for Fundamental Research and Development (973 Program, No. 2010CB950702)", "APN (Asia Pacific Network) Global Change Fund Project (No. ARCP2013-16NMY-Li)", the National Natural Science Foundation of China (No. 41271361), "The National High Technology Project (863 Plan, No. 2007AA10Z231)", and the Public Sector Linkages Program supported by Australian Agency for International Development (PSLP, No. 64828). The funders had no role in study design, data collection and analysis, decision to publish, or preparation of the manuscript.
