# Supplementary figures and images for: Assessing the Spatiotemporal Variation in Distribution, Extent and NPP of Terrestrial Ecosystems in Response to Climate Change from 1911 to 2000
Source: PLoS One. 2013 Nov 25;8(11):e80394. doi: 10.1371/journal.pone.0080394 (PMC3840029; doi:10.1371/journal.pone.0080394)

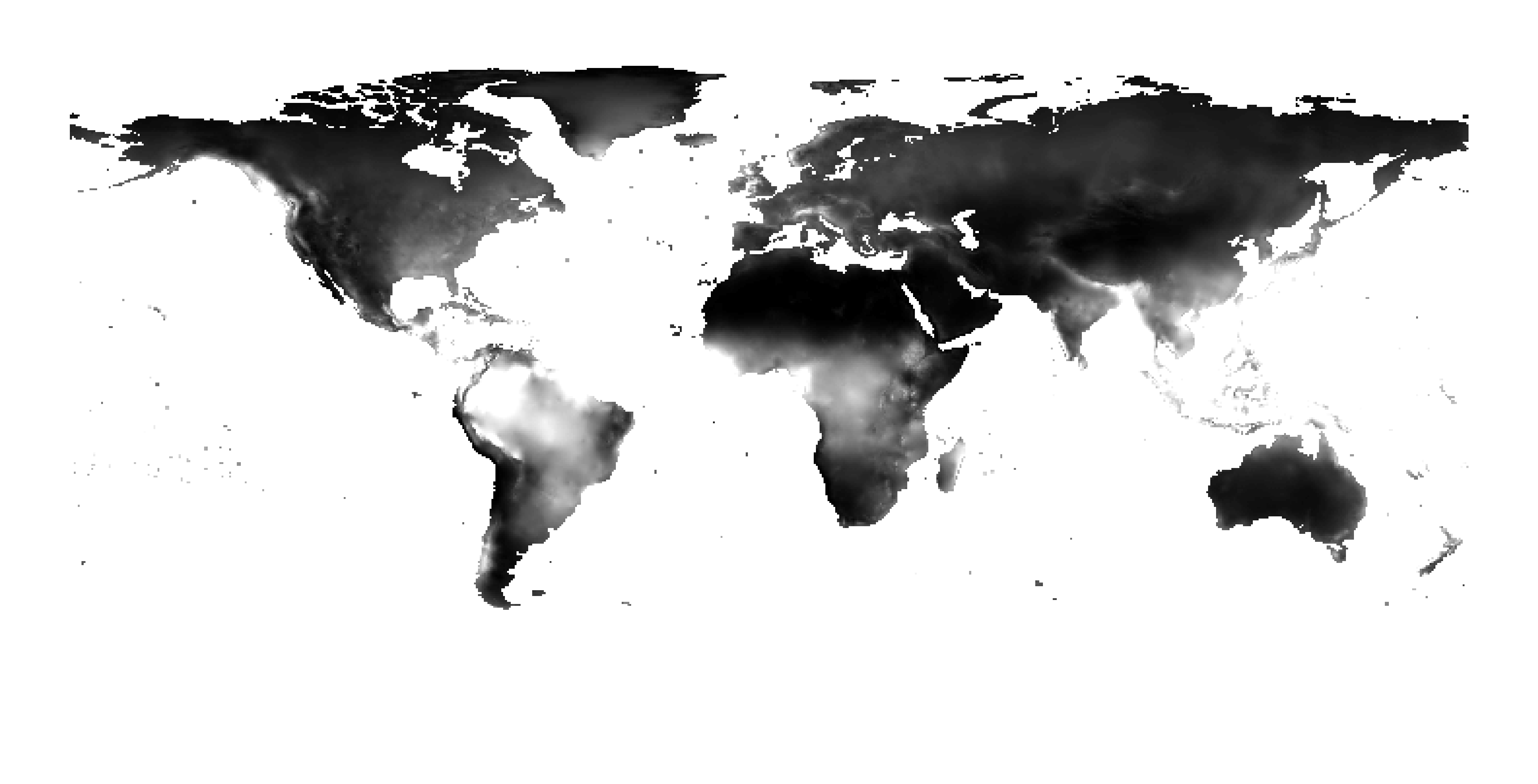

Supplement: Appendix S1 — MAP in T1 period. (TIF) [file pone.0080394.s001.tif]

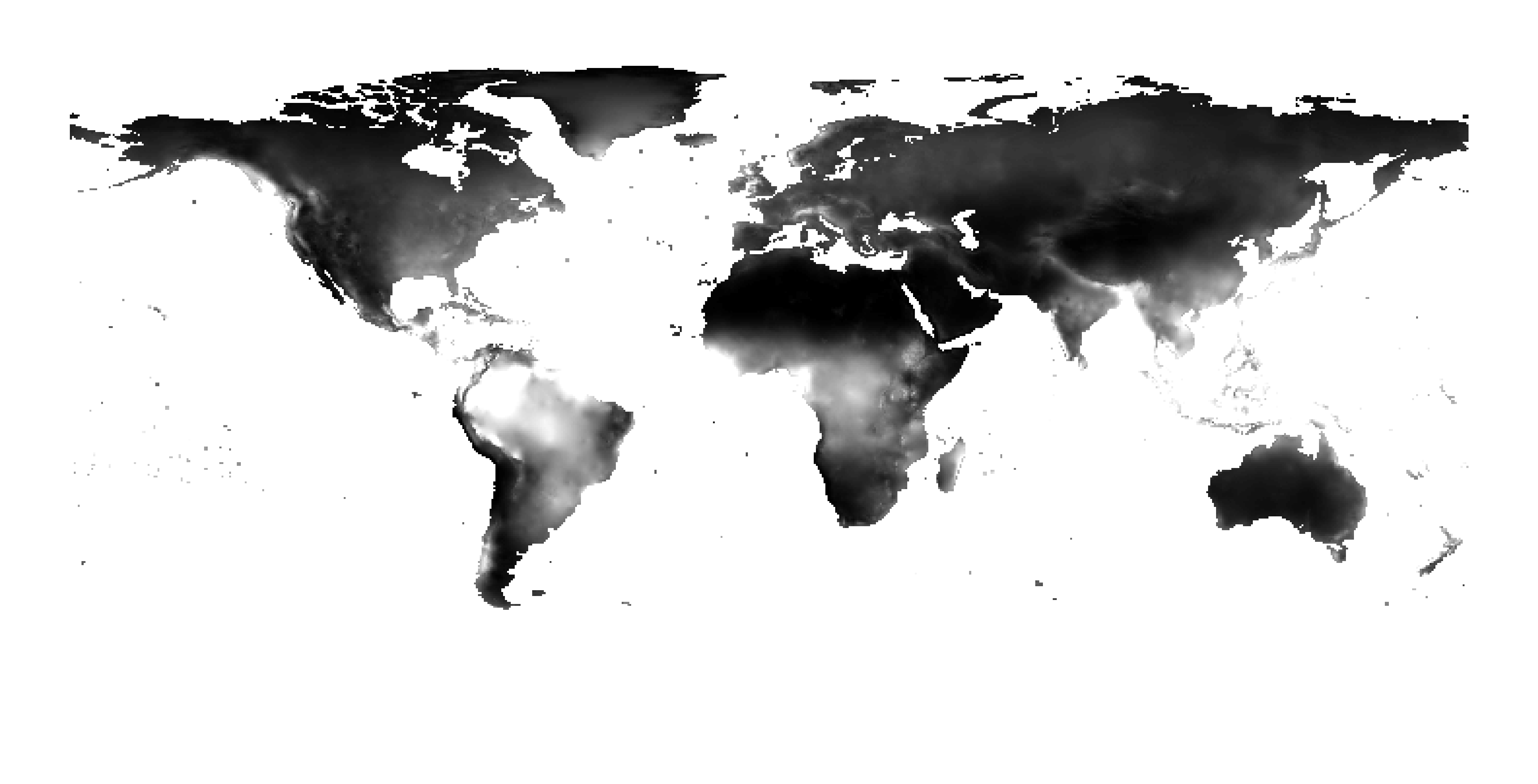

Supplement: Appendix S2 — MAP in T2 period. (TIF) [file pone.0080394.s002.tif]

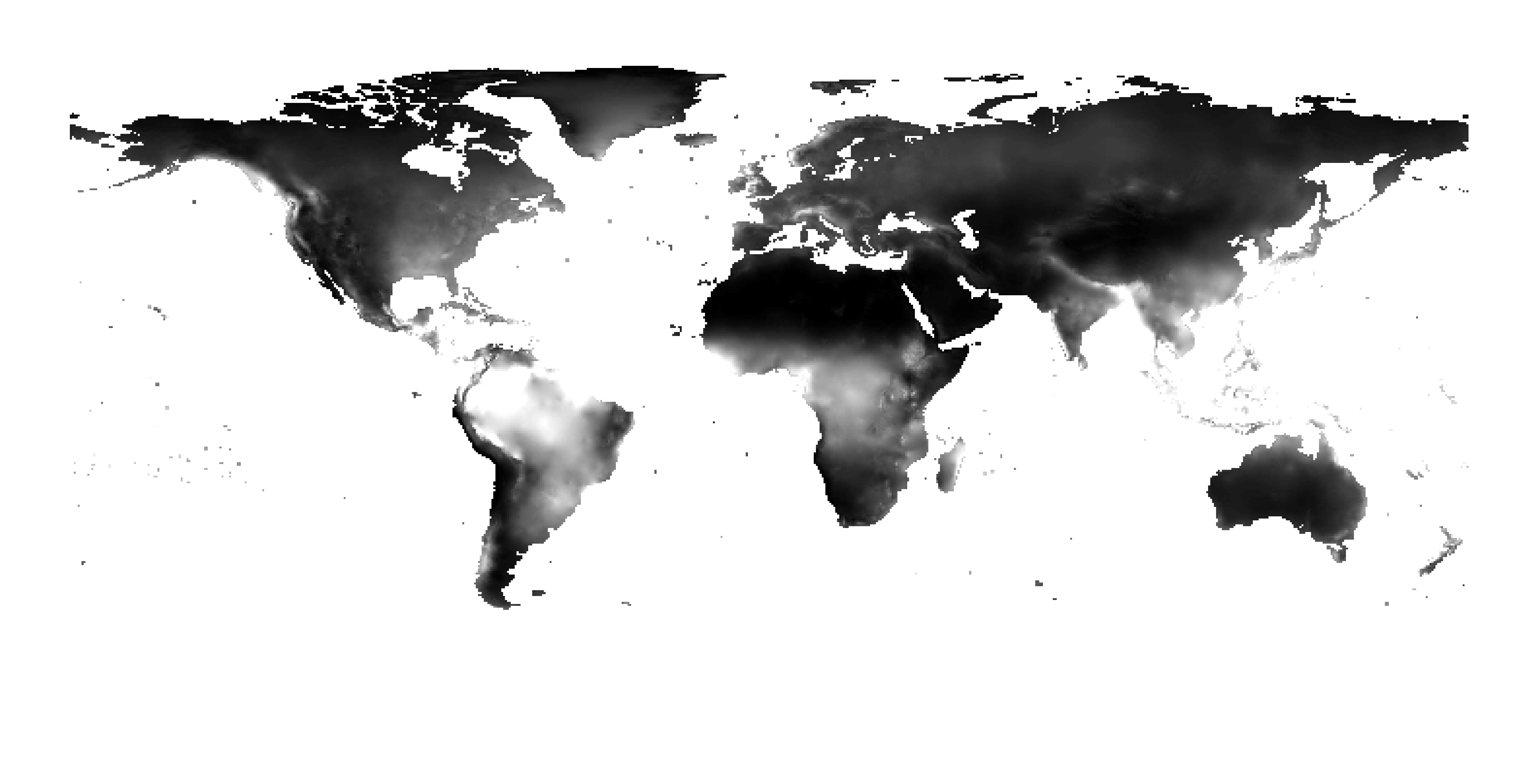

Supplement: Appendix S3 — MAP in T3 period. (TIF) [file pone.0080394.s003.tif]

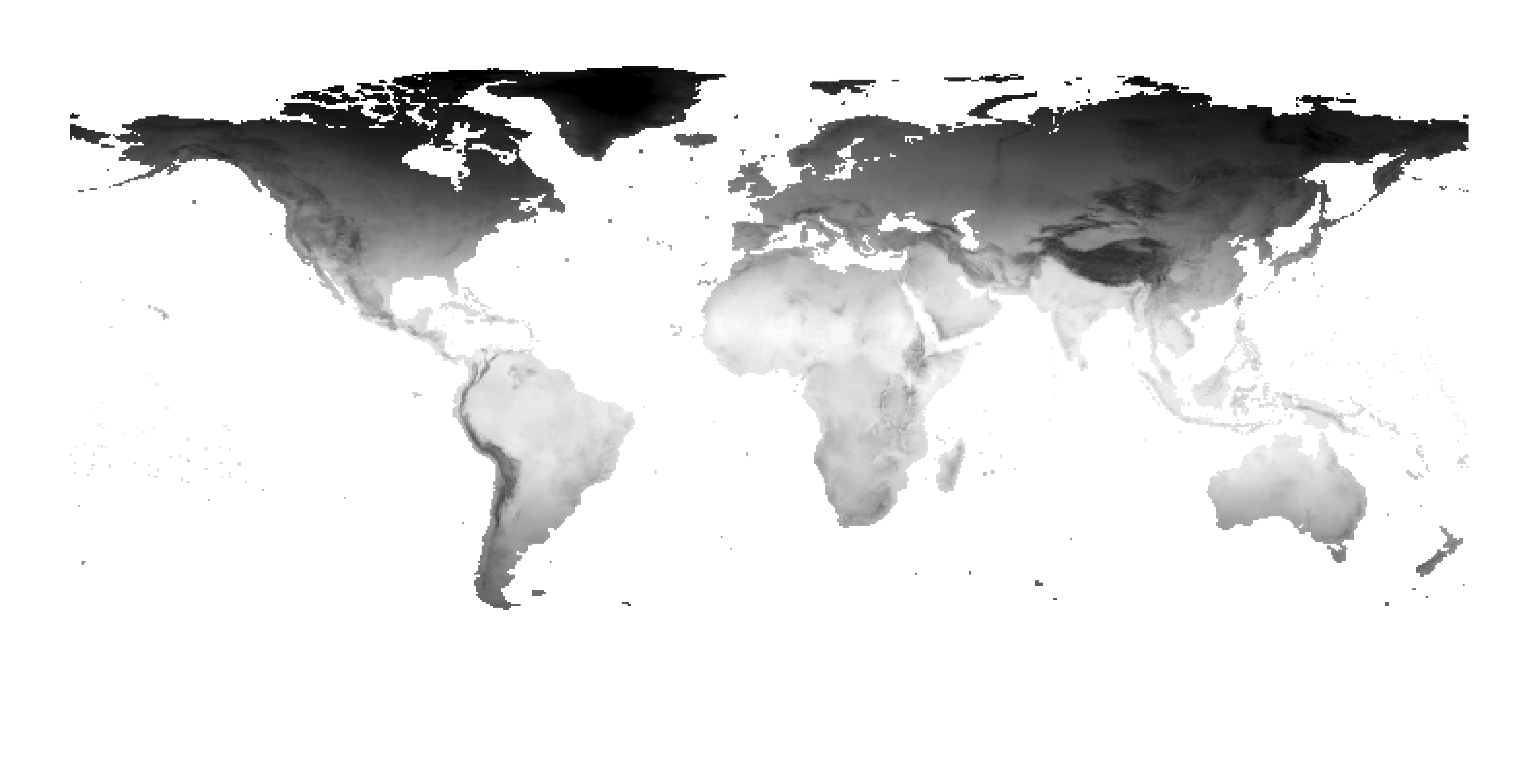

Supplement: Appendix S4 — MAT in T1 period. (TIF) [file pone.0080394.s004.tif]

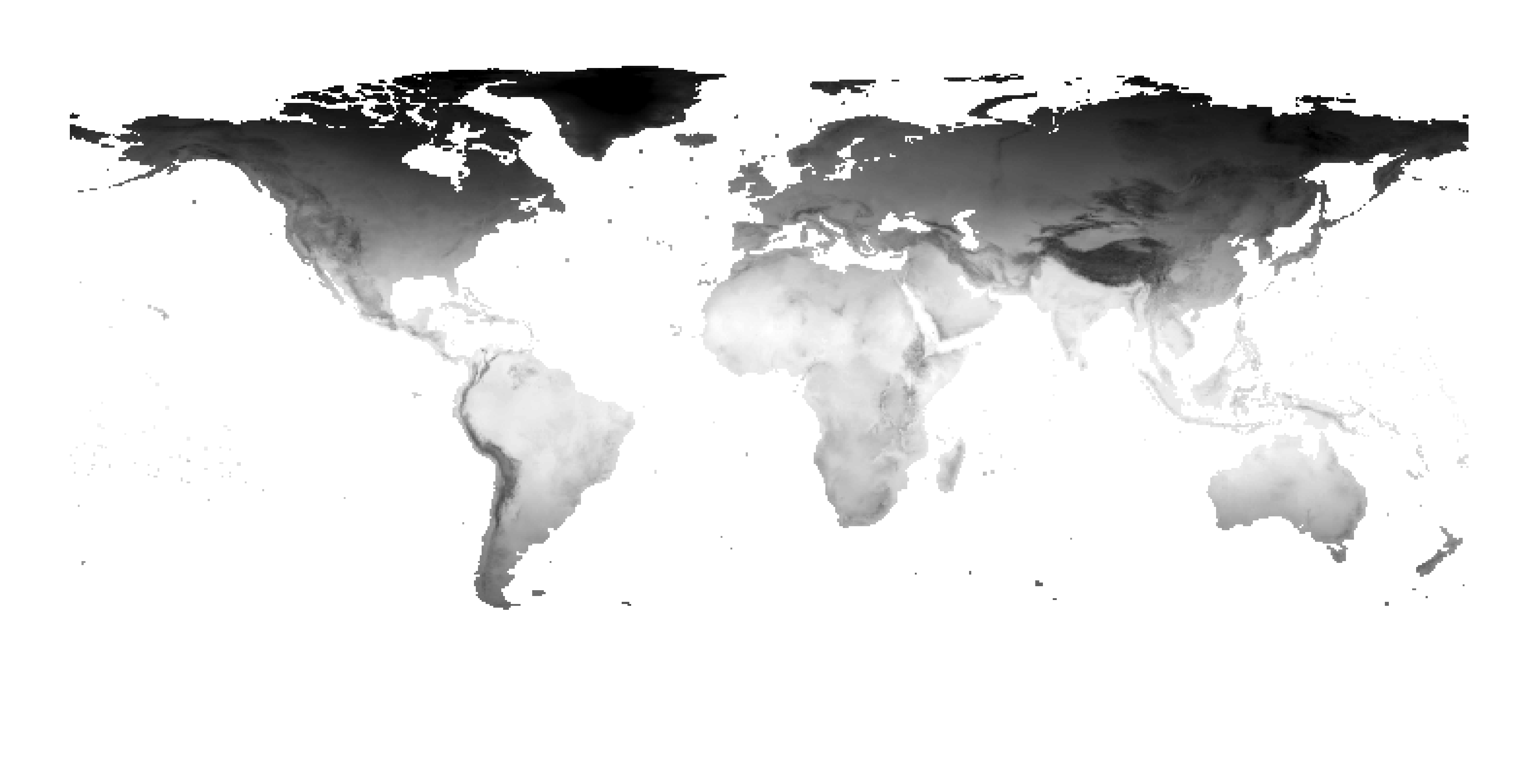

Supplement: Appendix S5 — MAT in T2 period. (TIF) [file pone.0080394.s005.tif]

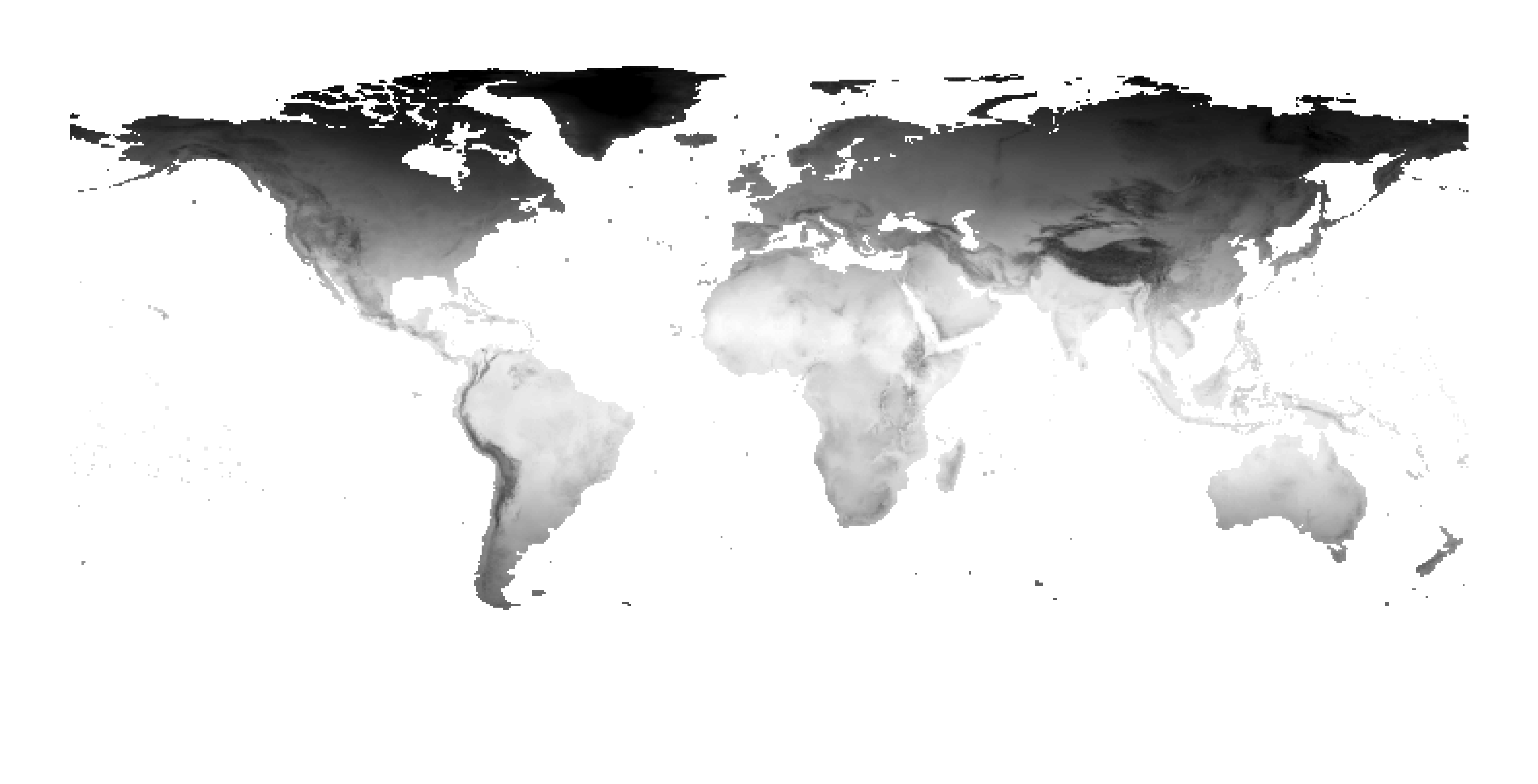

Supplement: Appendix S6 — MAT in T3 period. (TIF) [file pone.0080394.s006.tif]

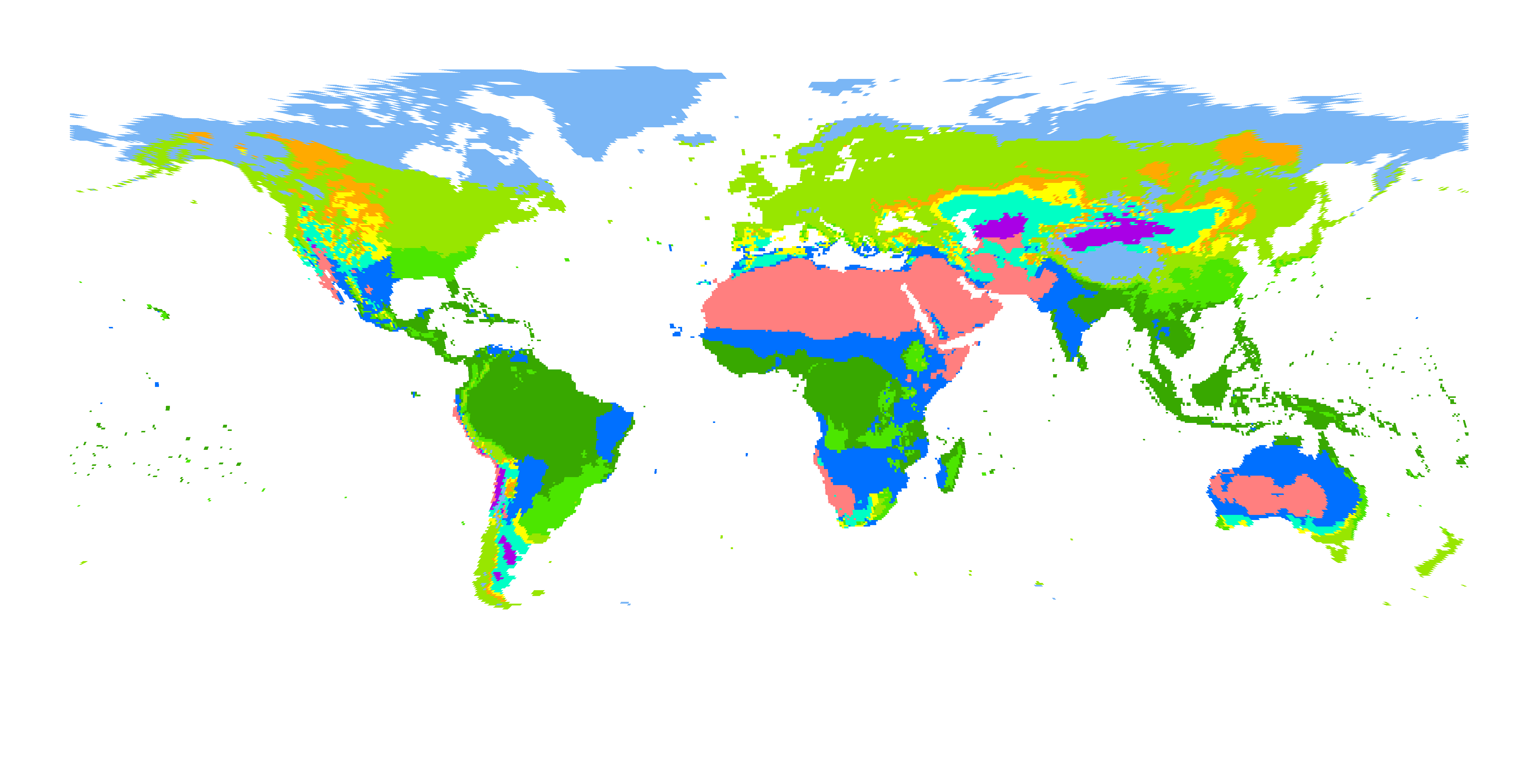

Supplement: Appendix S7 — The simulated global vegetation in T1 period. (TIF) [file pone.0080394.s007.tif]

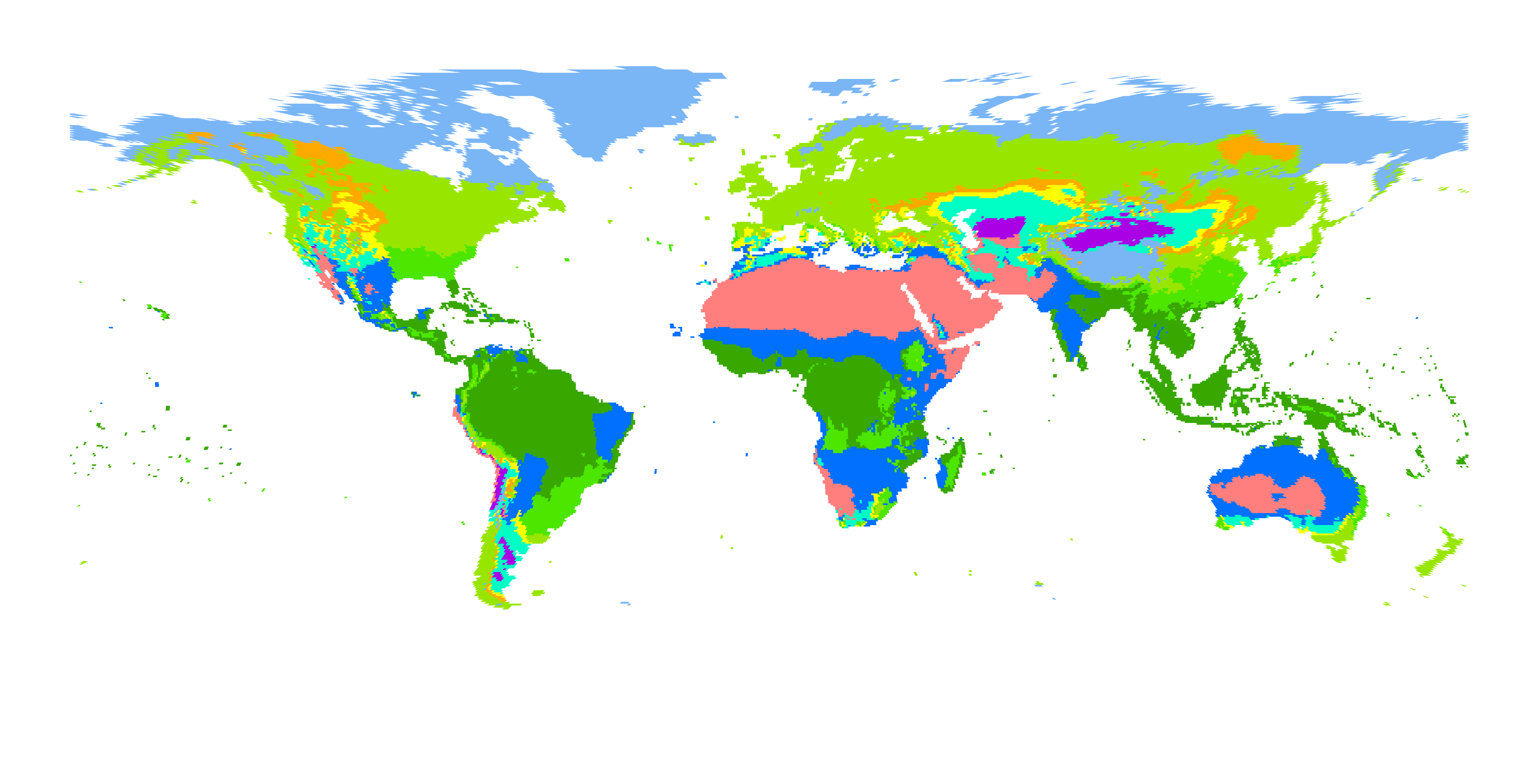

Supplement: Appendix S8 — The simulated global vegetation in T2 period. (TIF) [file pone.0080394.s008.tif]

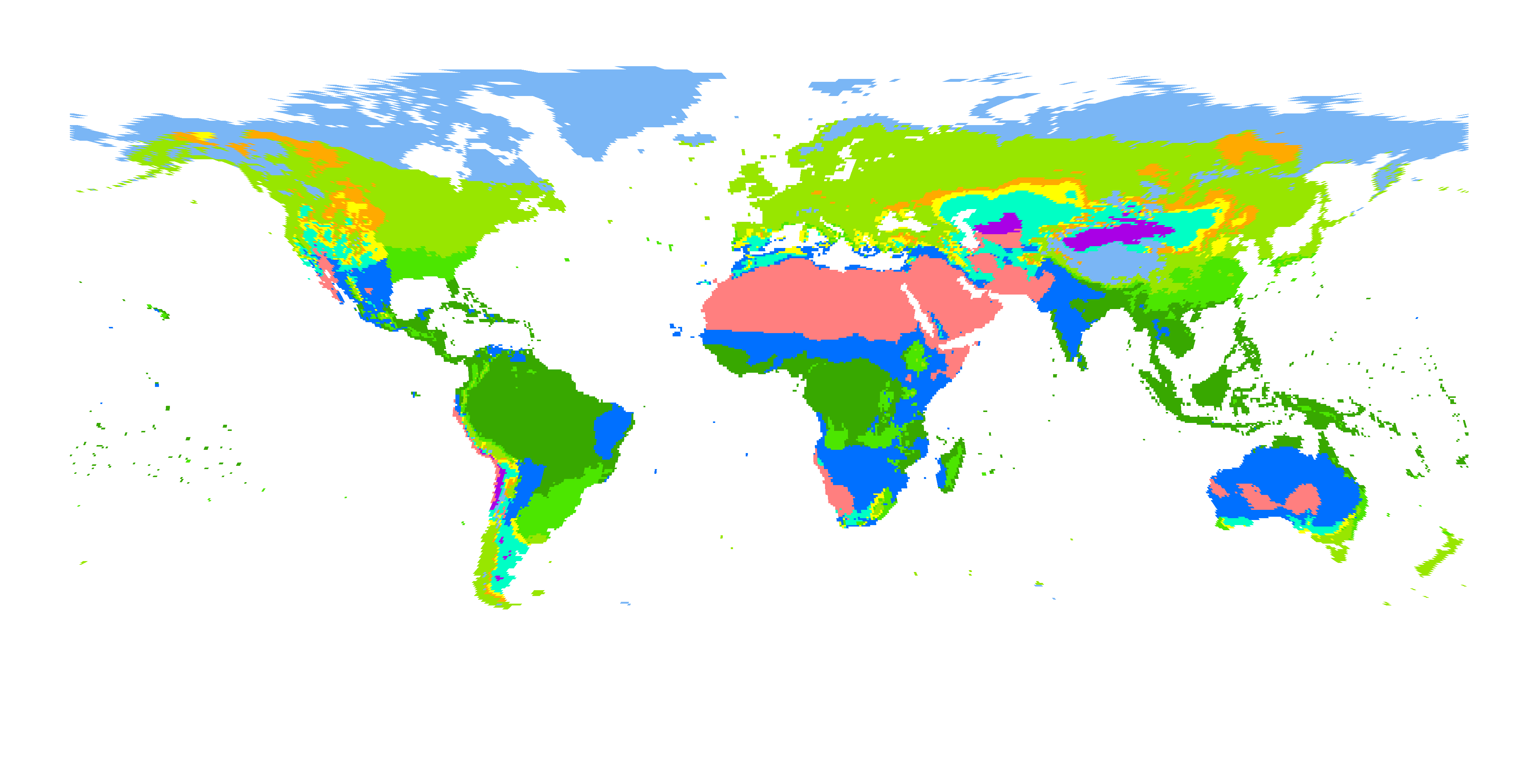

Supplement: Appendix S9 — The simulated global vegetation in T3 period. (TIF) [file pone.0080394.s009.tif]

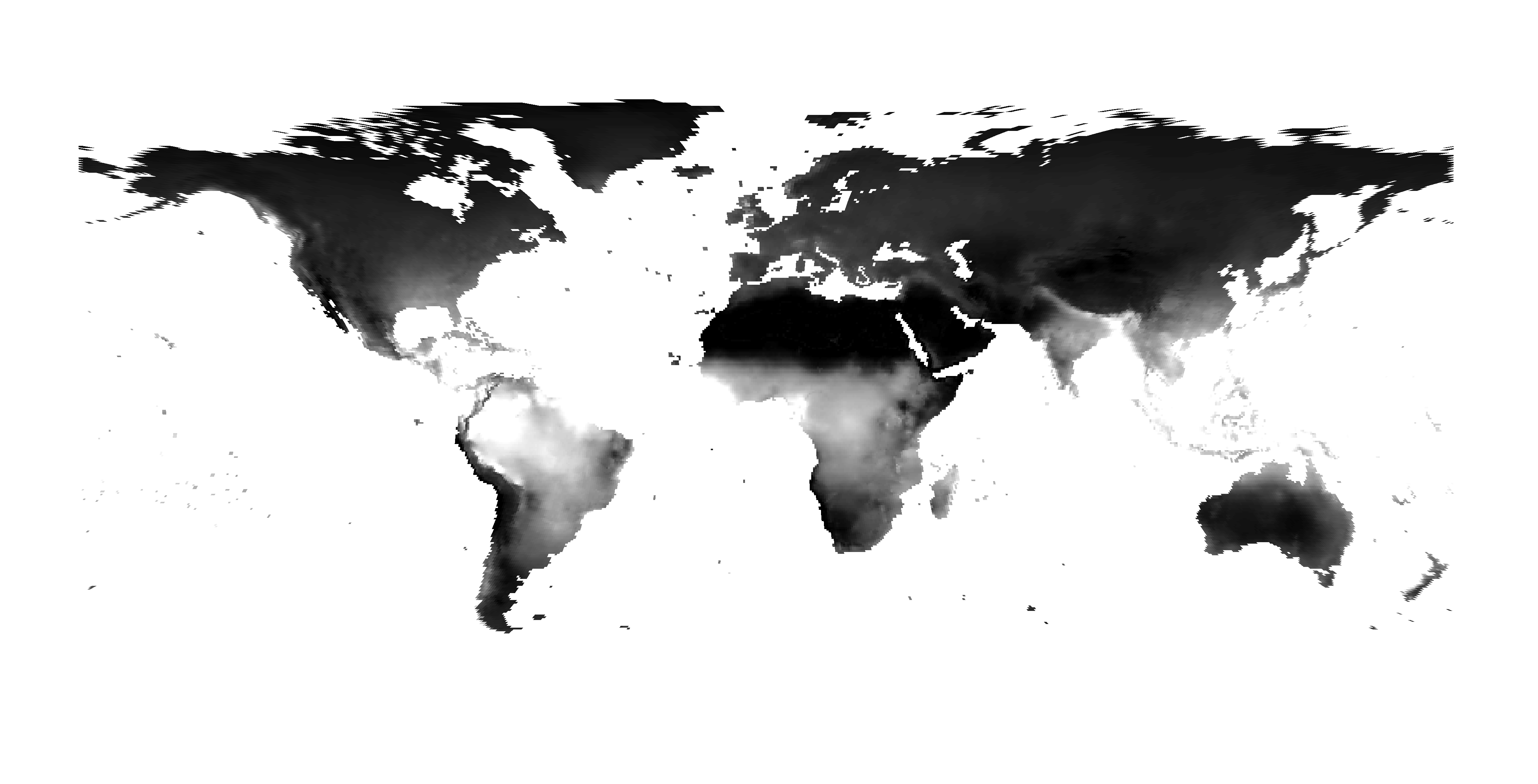

Supplement: Appendix S10 — Global NPP in T1 period. (TIF) [file pone.0080394.s010.tif]

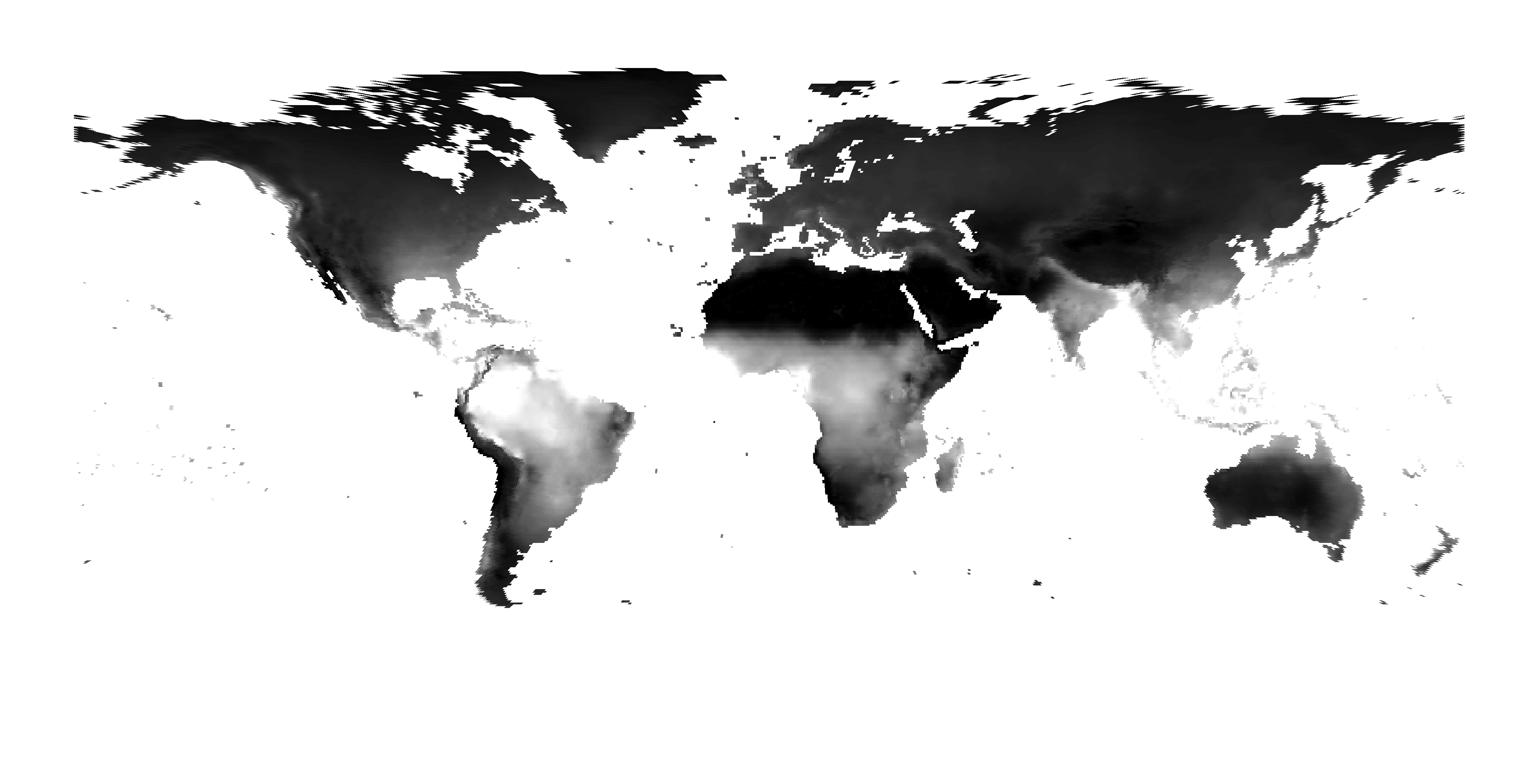

Supplement: Appendix S11 — Global NPP in T2 period. (TIF) [file pone.0080394.s011.tif]

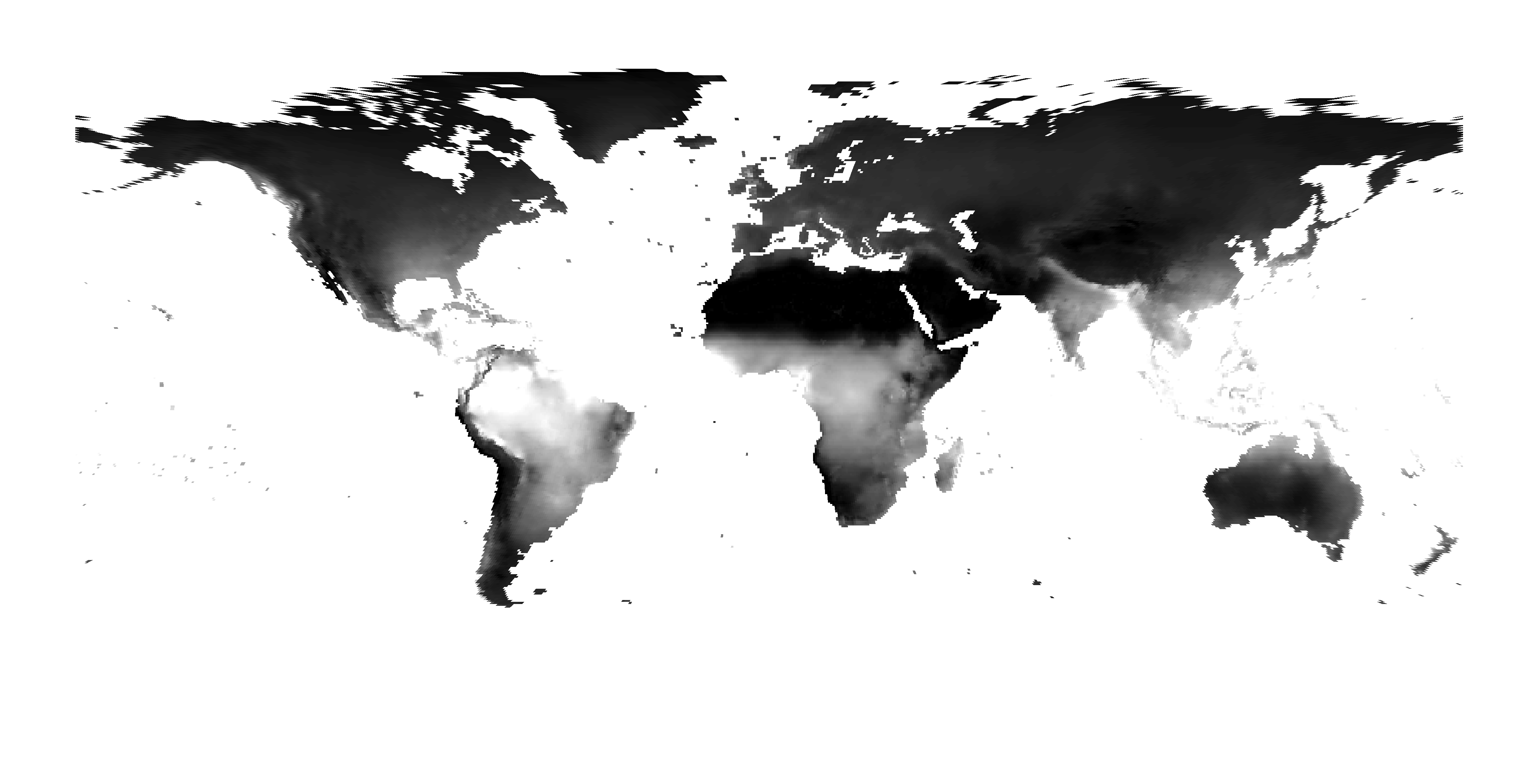

Supplement: Appendix S12 — Global NPP in T3 period. (TIF) [file pone.0080394.s012.tif]
